# Results of a European-Wide External Quality Assessment (EQA) Scheme for Serological Detection of Anti-SARS-CoV-2 (CoVimm)—Pitfalls of Routine Application

**DOI:** 10.3390/v14081662

**Published:** 2022-07-28

**Authors:** Maximilian Kittel, Romy Eichner, Sihem Aida, Anna Bode, Volker Ast, Anja Kessler, Michael Neumaier, Roman Wölfel, Verena Haselmann

**Affiliations:** 1 Department of Clinical Chemistry, University Medical Centre Mannheim, Medical Faculty Mannheim, University of Heidelberg, 68167 Mannheim, Germany; maximilian.kittel@umm.de (M.K.); romy.eichner@umm.de (R.E.); sihem.aida@umm.de (S.A.); volker.ast@medma.uni-heidelberg.de (V.A.); michael.neumaier@umm.de (M.N.); 2 Reference Institute for Bioanalytics, Stiftung für Pathobiochemie und Molekulare Diagnostik, 53175 Bonn, Germany; a.bode@spmd-rfb.de (A.B.); a.kessler@spmd-rfb.de (A.K.); 3 Bundeswehr Institute of Microbiology, 80937 Munich, Germany; romanwoelfel@bundeswehr.org; 4 German Center for Infection Research (DZIF), Partner Site Munich, 80333 Munich, Germany

**Keywords:** external quality control, SARS-CoV-2, test performance, external quality assessment scheme, proficiency testing, COVID-19, anti-SARS-CoV-2 antibodies, EQA, immunoassays, serological testing

## Abstract

Background: During the last two years, a variety of assays for the serological detection of antibodies to the new SARS-CoV-2 virus have been launched and used as part of standard care in many laboratories. The pace with which these tests have been introduced into routine care emphasizes the importance of quality measures for analytical methods, particularly with regard to the implications of results for clinical and epidemiologic decisions. Accuracy, reliability and comparability of analytical test results are thus essential, and here external quality assessment (EQA) is the most important quality assurance tool. It allows us to achieve harmonization of test methods as a prerequisite for a high standard of performance for laboratory and analytical techniques and their interpretation. Methods: This EQA scheme consisted of pre-characterized clinical biospecimens dedicated to the analysis of anti-SARS-CoV-2 IgG total antibodies and differentiation into spike protein-specific IgG antibodies against SARS-CoV-2 (anti-S-SARS-CoV-2) and nucleocapsid-specific IgG antibodies against SARS-CoV-2 (anti-N-SARS-CoV-2). Results: A total of 239 laboratories across Europe participated in this scheme, called CoVimm. In detail, 536 results for anti-SARS-CoV-2 IgG, 431 results for anti-S-SARS-CoV-2 IgG, and 200 results for anti-N-SARS-CoV-2 IgG were reported. Based on the pre-defined thresholds, the success rates for the determination of anti-S-SARS-CoV-2 IgG and anti-N-SARS-CoV-2 IgG were 96% and 90%, respectively. Interestingly, only 64% of the participating laboratories successfully passed the EQA scheme for the determination of total anti-SARS-CoV-2 IgG. Conclusions: This EQA revealed serious concerns regarding the reliability and appropriate use of anti-SARS-CoV-2 antibody assays in routine care. In addition to the wide heterogeneity of different assays used by participating laboratories, a lack of standardization and harmonization is also evident. This is of particular importance for reliable and clinically meaningful interpretation of test results.

## 1. Introduction

Detection of the immunological response to the SARS-CoV-2 virus infections is a cornerstone in the successful management of the ongoing pandemic. The gold standard of primary pathogen detection in suspected SARS-CoV-2 infections is the molecular genetic detection of at least two virus-specific gene loci by quantitative reverse transcription PCR (qRT-PCR) from respiratory material. Additionally, serologic testing is recommended for particular situations, such as patients with previous SARS-CoV-2 infections and patients with current infections who have presented symptoms for over three weeks [1,2,3,4]. Since the introduction of mRNA-based vaccination, serologic detection is used to evaluate the immune response or to differentiate between natural infection and vaccination [5]. Furthermore, serological assays facilitate accurately assessing the disease prevalence and its development, and they are furthermore used to evaluate the effectiveness of measures such as lockdowns, school closures, travel bans, and social distancing, thus emphasizing the tense nature of analysis results and their subsequent interpretation [6,7]. In addition to laboratory-developed assays, numerous major manufacturers have introduced test solutions for the detection of anti-SARS-CoV-2-specific antibodies to the market [8]. Not only the high-throughput automatization and applicability of existing analytical platforms, but also the great demand from the population have led to these tests being offered by a large number of laboratories.

These serological assays are capable of detecting different antibody classes, such as IgM, IgA, and IgG, as well as total antibodies. They also detect the target structure/viral epitope of the respective antibodies, e.g., the nucleocapsid, the spike protein, or the receptor-binding domain of the novel coronavirus. The most common COVID-19 antibody-detection methods in human serum or plasma include enzyme-linked immunosorbent assay (ELISA), chemiluminescence immunoassay (CLIA), electrochemiluminescence immunoassay (ECLIA), and fluorescence immunoassay (FIA). The assay design variables already indicate that large discrepancies in test results and their interpretations are to be expected between clinical laboratories [4,9].

The key instrument to ensure the highest possible standard of technical analyses and to achieve a harmonization of their results is an external quality assessment (EQA) [10,11]. This proficiency testing (PT) is a highly valuable quality-assurance factor in clinical laboratory and enhances the reliability of patient test results [12]. All participants in an EQA program blindly analyze distributed samples and report their test results and clinical evaluation to an approved and accredited provider within a specified time frame. In this context, it is important that these biospecimens are processed in the same way as routine samples. Subsequent evaluation allows for comparison of individual laboratory’s performance with the collective performance of all other participants or specific peer groups. In addition, the performance of the analytical methods used by the participants can be objectively evaluated, as well as the degree of harmonization between them. This approach can reveal discrepancies in result reporting between methods and manufacturers and thus play an important role in detecting variations in test performances and assess the current level of harmonization.

In the context of anti-SARS CoV-2 serological proficiency testing, the Reference Institute for Bioanalytics (RfB) was the first EQA provider worldwide to pilot in April 2020 [13]. In this manuscript, the outcomes of the latest conducted EQA scheme, briefly called CoVimm, are reported with the aim of providing an overview of currently used anti-SARS-CoV-2 serological assays, offering insights into their diagnostic test performance, and revealing the variability of interpretation of test results.

## 2. Materials and Methods

### 2.1. Study Design

The CoVimm EQA scheme was first conducted as pilot in April 2020 on behalf of the COVID-19 Task Force of the German Society for Clinical Chemistry and Laboratory Medicine [13]. After successful implementation of this scheme, the EQA was established as a routine program and offered semi-annually. The RfB is a DIN EN ISO/IEC 17043:2010 accredited EQA provider.

Within the scheme a panel of samples were provided to the participating laboratories. The panel consisted of four pre-characterized, pseudonymized serum samples from volunteers and was dedicated to the analysis of total anti-SARS-CoV-2 IgG, anti-N-SARS-CoV-2 IgG, and anti-S-SARS-CoV-2 IgG antibodies. The study was conducted in accordance with the Declaration of Helsinki. Each subject’s medical history was obtained with the use of digitalized, standardized questionnaires (REDCap, Version 10, Vanderbilt University, Nashville, TN, USA). All participants were recruited at the University Hospital Mannheim, Germany, as part of the Immunitor-Study. The study was approved by the institutional review board (registration number: 2020-556M), and written consent was obtained from each participant before sample collection. The positive samples included patient sera with different anti-SARS-CoV-2 antibody titers. The pre-characterization of the samples was conducted by two organizing institutions: the Institute for Clinical Chemistry at the University Medical Centre Mannheim at the Medical Faculty Mannheim, University of Heidelberg and Bundeswehr Institute of Microbiology, Munich as described previously [14].

Each participating laboratory received four 600 μL samples of human serum for COVID-19 antibody detection. These were accompanied by a cover letter giving a clinical background, basic instructions, and a report template. The samples were centrally distributed by the RfB to the participants by using a logistics service provider at ambient temperature on 15 October 2021. The closing date for submitting the results was 30 October 2021. Participants were instructed to use their standard procedures to assess the anti-SARS-CoV-2 antibody class and report qualitative results (positive, negative, or borderline). All results were assessed by the RfB.

The pre-defined minimum criteria for successful performance were as follows: (a) correct identification of samples provided for the particular target (e.g., total antibodies, anti-N-antibodies, anti-S-antibodies) and (b) correct analysis of all samples. Participants were eligible to submit results for separate test systems or kits, but it was mandatory to agree on a final assessment for each sample (i.e., positive, negative, or borderline). This served as the basis for passing the proficiency test and issuing of certificates. A summary report of statistics and final individual results were sent to all participants and is available at the RfB website [15]. Certificates of anti-SARS-CoV-2 serologic testing were also distributed for each correctly analyzed target (e.g., total antibodies, anti-N-antibodies, anti-S-antibodies).

### 2.2. Sample Preparation

The EQA samples were prepared as previously published and described in the EQA standard operating procedures [14,16,17].

Briefly, after blood collection, serum samples were stored upright at room temperature for at least 1 h to allow for adequate clotting. Afterwards the samples were centrifuged for 10 min at 2000× *g* at 18 °C within 4 h of sample collection. The supernatants from the separate tubes were then pooled within the subject. This serum pool was transferred to cryotubes (LVL technologies GmbH & Co., KG, Crailsheim, Germany) and stored at −80 °C. In parallel, in order to avoid freeze–thaw cycles, 600 μL aliquots were prepared for pre-characterization of samples and stored at −80 °C. The day before shipment, the remaining serum pool was thawed, the different antibody classes were verified by analyzing at least seven different aliquots. The serum pool was shipped to the RfB by express delivery, was aliquoted and distributed to the participating laboratories. Additionally, some aliquots were kept and analyzed at predefined timepoints within the next two weeks in order to evaluate and ensure sample stability.

### 2.3. Laboratory Characterization of EQA Samples

Before dispatch, the two organizing institutes separately evaluated the four EQA samples with different immunoassays for anti-SARS-CoV-2-specific anti-S and anti-N IgG and total IgG antibodies, as well as for the ability to neutralize the virus by a virus-neutralizing-antibody-test (micro-VNTs).

SARS-CoV-2 antibodies were analyzed with the cobas e601 analyzer (Roche Diagnostics, Mannheim, Germany) using the Elecsys^®^ anti-SARS-CoV-2 electrochemiluminescence immunoassay-N for the qualitative detection of SARS-CoV-2 antibodies in plasma or serum. The Elecsys^®^ assay employs a modified double-antigen sandwich immunoassay using recombinant nucleocapsid protein (N) designed to detect high-affinity antibodies regardless of subclass. It is an assay for all SARS-CoV-2 antibodies (IgA, IgM, and IgG) that predominantly, but not exclusively, detects IgG. The results are reported as numerical values in the form of a cut-off index (COI; signal sample/cutoff) and in the form of non-reactive (COI < 1.0; negative) and reactive (COI ≥ 1.0; positive) qualitative results.

Based on the identical principle, the anti-SARS-CoV-2-S Elecsys^®^ assay (Roche Diagnostics, Mannheim, Germany) was used. This double-antigen sandwich immunoassay targets the spike protein of the novel coronavirus, on which all mRNA vaccines currently available are based. The assay also detects all SARS-CoV-2 antibodies (IgA, IgM, and IgG) and provides quantitative measurement results.

These measurements were performed in a series of 5 measurements, and their mean value was used.

The Euroimmun Anti-SARS-CoV-2 Assay (EUROIMMUN AG, Lübeck, Germany) is an enzyme-linked quantitative in vitro assay for the determination of human antibodies of the IgG immunoglobulin class against SARS-CoV-2. The manufacturer also offers different assays for the specific detection of antibodies against the nucleocapsid and the spike protein. Both assays are based on microtiter strips coated with the recombinant structural protein of SARS-CoV-2. After the incubation of the patient sample followed by a washing step, anti-SARS-CoV-2-specific antibodies bind to the coated antigens in the case of positive samples. Detection is performed by a second incubation with an enzyme-labeled anti-human IgG (enzyme conjugate) that induces a color reaction throughout catalysis. The results are semi-quantitatively expressed as a ratio to the calibrators as follows: <0.8 negative; ≥0.8 to <1.0 borderline; ≥1.1 positive.

The virus micro-neutralization tests (micro-VNTs) were performed under biosafety level 3 conditions. Serum samples were heat-inactivated at 56 °C for 30 min. Subsequently, the assay was performed with 50 μL in serial dilutions ranging from 1:10 to 1:80 in duplicates. Virus stock solutions were used versus wild-type and delta-variant virus. After incubation for one hour at 37 °C, a 50 μL Vero-E6 cell (2 × 10^5^ cells per mL) suspension was added to each batch. These suspensions were incubated for 72 h at 37 °C and 5% CO^2^. Subsequent evaluation was microscopically performed using 0.1% crystal violet staining solution. The titer of the respective serum was determined by the highest concentration at which complete virus neutralization took place. Positive and negative patient sera were used as controls.

All assays were used according to manufacturers’ instructions, and all obtained results are summarized in Table 1. Results were analyzed in combination with the patients’ clinical data and medical history by a panel of experts, and each sample was assigned target values in this regard.

### 2.4. Sample Characteristics

The following detailed clinical information was compiled for the patient samples that were used in the EQA scheme:

**Sample 1:** 34-year-old asymptomatic patient who tested positive for SARS-CoV-2 by qRT-PCR and was treated as an outpatient. Blood sampling was performed 50 days after infection. Spike-protein-specific IgG antibodies against SARS-CoV-2 (anti-S-SARS-CoV-2) without neutralizing activity were detected. No nucleocapsid-specific IgG antibodies against SARS-CoV-2 (anti-N-SARS-CoV-2) were detected.

**Sample 2:** 65-year-old symptomatic patient who tested positive for SARS-CoV-2 by qRT-PCR and was treated as an outpatient. Blood sampling was performed 238 days after infection. Spike-protein-specific IgG antibodies against SARS-CoV-2 (anti-S-SARS-CoV-2) without neutralizing activity were detected in the diluted sample (1:1.3) dispatched in this EQA. Furthermore, nucleocapsid-specific IgG antibodies against SARS-CoV-2 (anti-N-SARS-CoV-2) were detected.

**Sample 3:** SARS-CoV-2-negative patient pool consisting of sera from healthy blood donors with no clinical evidence of SARS-CoV-2 infection in the past. Antibodies were not detected by the use of various immunoassays or the micro-VNTs.

**Sample 4:** 38-year-old control patient with no clinical evidence of a SARS-CoV-2 infection in the previous five months. The patient was vaccinated twice with a mRNA vaccine. Blood sampling was performed 87 days after the second vaccination. Spike-protein-specific IgG antibodies against SARS-CoV-2 (anti-S-SARS-CoV-2) with neutralizing activity against wildtype SARS-CoV-2 but without neutralizing activity against the delta virus variant were detected. No nucleocapsid-specific IgG antibodies against SARS-CoV-2 (anti-N-SARS-CoV-2) were detected.

### 2.5. Statistical Analysis

All quantitative and qualitative results reported to the EQA provider were evaluated according to the pre-defined thresholds. Only results reported for the respective antibody class were considered to quantify the error rate. For the method-specific error rate, only results of those laboratories using that particular method were evaluated. Data analysis is presented in the form of descriptive statistics, including sensitivity, specificity, and the respective 95% confidence interval (95% CI). All statistical analyses and graphical representations were performed using the jamovi project^TM^ (Version 1.6) and Prism 7 (Version 7, GraphPad, San Diego, CA, USA).

## 3. Results

### 3.1. Participation and Scope

A total of 211 laboratories from 17 countries participated in this EQA scheme. The majority of participants (*n* = 179; 84.8%) originated from Germany, followed by Austria (*n* = 7; 3.32%), the Netherlands with (*n* = 4; 1.9%), and Switzerland (*n* = 4; 1.9%). A total of 1276 measurement results for the determination of anti-SARS-CoV-2 antibodies were submitted by the participants, though not all participated in all three analytical procedures.

For the task of total anti-SARS-CoV-2 IgG antibody detection, a total of 624 results were submitted by 129 different laboratories, and 49% of the total submitted results are attributable to this section of the EQA. A total of 27 contributing laboratories accepted the offer to submit their results for multiple analysis platforms. Analytical platforms from 20 different manufacturers were used, with majority of laboratories using the vendor Roche (*n* = 212; 34%), followed by DiaSorin (*n* = 104; 17%) and Euroimmun (*n* = 96; 15%).

For the second task, the determination of anti-S-SARS-CoV-2 IgG antibodies, a total of 447 results were submitted by 100 participating laboratories. Accordingly, 35% of all submitted analytical results are attributable to this section of the EQA. Twelve participants submitted measurement results for more than one analytical platform. The majority of participating laboratories used instruments from the vendor Roche (*n* = 175; 39%), followed by Euroimmun (*n* = 76; 17%) and Abbott (*n* = 56; 13%).

The fewest participants performed the last part of the EQA, the determination of anti-N-SARS-CoV-2 IgG antibodies. A total of 204 measurement results were submitted by 47 participants. Only 16% of the submitted results covered this section of the EQA. Four participants submitted results for more than one analytical platform. The majority of the participating laboratories also used platforms from the vendor Roche (*n* = 104; 51%), followed by Abbott (*n* = 44; 22%) and Euroimmun (*n* = 20; 10%). For this EQA part, detection systems from 23 different vendors, as well as lab-developed test systems were used.

In total, 61.14% of all participating laboratories reported results for total SARS-CoV-2 IgG antibody detection, 47.39% for anti-S-SARS-CoV-2 IgG detection and 22.27% for anti-N-SARS-CoV-2 IgG analysis.

### 3.2. Success Rate and Sample-Specific Error Rate

The overall performance was evaluated according to the previously defined criteria mentioned above. The target values for each EQA sample are summarized in Table 2 and Table 3.

Among the 624 results submitted for evaluation, a total of 78.96% (*n* = 491) of total anti-SARS-CoV-2 IgG antibodies were classified as correct, 1.12% (*n* = 7) were classified as borderline, and 20.19% (*n* = 126) were classified as incorrect in terms of predefined results. Samples 1, 2, and 4 were considered positive, with borderline test results rated as conditionally correct. On the basis of this evaluation scheme, 82/129 laboratories correctly analyzed all four samples for total anti-SARS-CoV-2 IgG, which resulted in a success rate of 64%, a false-negative rate of 20.2%, and a false-positive rate of 3.7%. Particularly, the false-negative rate was strikingly high.

Of the 447 results submitted by the 100 participants for the detection of anti-S-SARS-CoV-2 antibodies, a total of 95.3% (*n* = 426) were classified as correct, 0.67% (*n* = 3) were classified as borderline, and 4.03% (*n* = 18) were classified as incorrect with respect to the predefined expected results. Samples 1, 2, and 4 were classified as positive, and the borderline test results were considered as conditionally correct. Samples 1 and 2 were from patients who had undergone proven SARS-CoV-2 infection, and sample 4 was from a healthy donor who had been vaccinated twice with an mRNA vaccine. Based on this evaluation scheme, 96/100 laboratories correctly analyzed all four samples for anti-S-SARS-CoV-2 antibodies, which resulted in a success rate of 96%. In this section of the EQA, the false-negative rate was 0.9% and the false-positive rate was 1.8%.

Out of the 204 results submitted by the 47 participants for detection of anti-N-SARS-CoV-2 IgG antibodies, a total of 72.55% (*n* = 148) of the results were considered correct, 0.98% (*n* = 2) were considered borderline, and 26.47% (*n* = 54) were considered incorrect. Samples 1, 3, and 4 were scored as negative, and sample 2 was scored as positive, with borderline also scored as correct and negative test results scored as conditionally correct. Sample 2 involved a patient who presented symptomatic SARS-CoV-2. Due to the time lag between blood sampling and infection, as well as the lack of evidence of a neutralizing effect of the detected anti-SARS-CoV-2 antibodies, all submitted test results were evaluated as conditionally correct (Table 1). Based on this evaluation scheme, 43/47 laboratories correctly analyzed all four samples for the presence of anti-N-SARS-CoV-2 IgG antibodies to novel coronavirus, which resulted in a success rate of 90% and a false-negative rate of 3.9%; a false-positive rate could not be defined.

The diagnostic performance for the detection of total anti-SARS-CoV-2, anti-S-SARS-CoV-2, and anti-N-SARS-CoV-2 IgG antibodies was evaluated for all assays that were used by the participants; these results are shown in Table 2. Furthermore, diagnostic sensitivities and specificities were provided together with their respective 95% CI as far as the sample selection would allow. For some assays, values of 100% were obtained for diagnostic specificity and sensitivity. It should be noted that mostly only a small number of results were provided for these assays.

Significant differences between the individual manufacturers were revealed, particularly for anti-SARS-CoV-2 total antibody detection. Considering the five most commonly used manufacturers in respect to detection of total anti-SARS-CoV-2 IgG antibodies, DiaSorin and Euroimmun showed the best performance with diagnostic sensitivities of 96% and 92%, respectively, and sensitivities of 88% and 92%. The best diagnostic performance was obtained for the Abbott assay with a diagnostic sensitivity of 72% and a diagnostic specificity of 100%, indicating that this assay could be used for routine diagnostics regarding its diagnostic specificity. For some other assays with even more promising results, the number of participants using these methods is not sufficient to evaluate and assess the diagnostic performance confidently in the context of routine clinical care.

**Figure 1:**
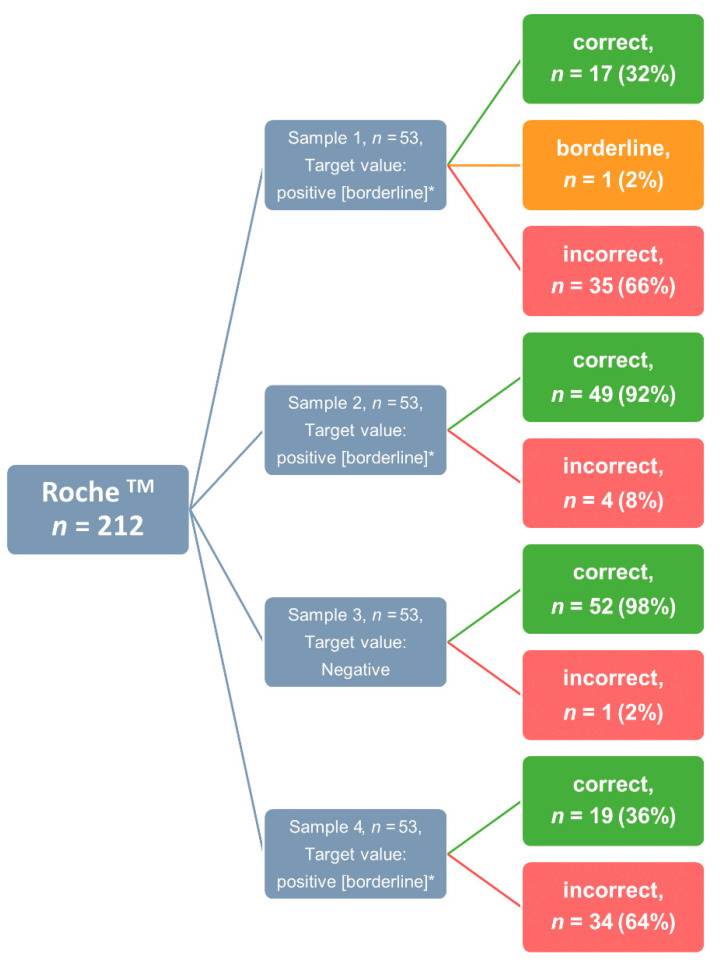
Distribution of results for the manufacturer Roche, for the subtask of total anti-SARS-CoV-2 IgG determination. The number of correct results (green), borderline results (orange), and incorrect determinations (red) are displayed per sample. Abbreviations: * = these results were considered as correct for total evaluation.

Interestingly, the 53 laboratories that reported using a Roche assay reported false results in 35% of the cases (*n* = 74). This phenomenon was in particular observed in samples 1 and 4, with a very high fraction of false negative results (Figure 1, Table 4). In a comparison of the five most commonly used manufacturers for the determination of anti-S-SARS-CoV-2 antibodies, DiaSorin and Siemens Healthineers showed by far the best performance with a diagnostic sensitivity and specificity of 100%, limited by the fact that only 20 and 36 participants used these tests, respectively. Roche’s assay showed a high diagnostic sensitivity of 95% with a specificity of 100% in a large number of participants.

When considering the five most commonly used manufacturers for the determination of anti-N-SARS-CoV-2 antibodies, it was unfortunately not possible to provide any information on diagnostic sensitivity due to sample selection. For all test kits used, with exception of that from Roche, a specificity of 100% was achieved.

## 4. Discussion

The ongoing pandemic has not only led to widespread public awareness of diagnostics, but also to considered discussions about the limitations of the validity of test results [16,17]. Most notably, serological testing has been used to evaluate the effectiveness of measures, some of which involved high personal and economic constraints, such as school, retail and gastronomy closures [18,19].

The assumption that it is better to use an inferior test method than none at all is simply wrong. The multiple consequences that can result from poorly performing assays were recently addressed by Gray et al., who used SIR modeling to demonstrate that false-positive and false-negative results will accumulate even with high-precision assays when performed as mass screenings. The authors outlined the impact and magnitude of misdiagnosis associated with ending the ban [20].

Since the onset of the pandemic and the availability of antibody tests, there have been insistent warnings that interpretation of test results might be challenging and must be done in the context of the ongoing pandemic [21]. Due to the prolonged low prevalence, the significance of antibody tests in terms of their positive-predictive value was limited. The consequences of false-negative and false-positive test results, which could lead to an over- or underestimation of the pandemic situation and the measures taken, have been extensively discussed in this context, especially before the emergence of the delta variant and the resulting rapid increase in prevalence [22]. As a result of the increased prevalence, the discussion regarding the diagnostic value of the positive predictive value has also subsided [23,24,25]. In any case, however, the reliability of serological test results in terms of high diagnostic sensitivity and specificity is a prerequisite for the inclusion of serological tests in clinical decision-making, as is the correct use and interpretation of test results. From the results of this EQA scheme, it can be deduced that there are distinct user-side issues in the correct application of the test procedures. An example would here be the misapplication of the Roche assay for the detection of total anti-SARS-CoV-2 IgG. This matter will be discussed in detail in the following section. Additionally, it is worth mentioning that the performance of the test procedures under real-world conditions (as seen in EQA schemes) was substantially worse than under study conditions or according to manufacturer’s claims. For example, Abbott reports a sensitivity of 99.37% for its SARS-CoV-2 IgG II quant assay specifically designed to detect the spike-RBD-based vaccine response [26]. This is remarkable higher than the 93% diagnostic sensitivity achieved by the Abbott cohort in this EQA. That the use of laboratory tests under so-called real-world conditions is inferior to the manufacturer claims has already been adequately proven by other authors for SARS-CoV-2-specific antibodies as well as for HIV testing [27,28].

EQA testing is probably the most important instrument for assuring quality in laboratory medicine, especially since the spectrum of EQAs includes analytical performance and pre- and post-analytical components [29]. It is used to verify at regular intervals whether laboratory results meet the quality requirements for patient care. This function of EQA has become even more important since the U.S. Department of Health and Human Services created the possibility of emergency use authorization (EUA) for laboratory tests related to SARS-CoV-2 diagnostics in February 2020. This enables the Food and Drug Administration (FDA) to grant EUA for a product if, after reasonable consideration, it can be expected to be effective [30]. That EQA providers have taken this responsibility seriously is evident from the short time to availability. For example, the first U.S. citizen was diagnosed with the novel coronavirus by PCR on 21 January 2020, and the first EQA samples were dispatched on 6 April 2020 [31,32]. Proficiency testing programs regarding indirect pathogen identification by means of SARS-CoV-2-specific antibody detection were made available even faster. The first EQA samples were dispatched as early as 28 April 2020 by the RfB in the framework of a pilot EQA [13].

The number of participants in this EQA demonstrates that there is a constant demand for serological assays, particularly to assess antibody response after vaccination. It is remarkable that automated assays were nearly exclusively used and only a few results for POC (point of care) tests were reported. It might be assumed that the increasing demand for assays to detect antibodies against SARS-CoV-2 by patients and senders has led to these being offered by laboratories. This hypothesis is strongly supported by the observation that the number of participants in the proficiency testing first steadily increased and then shifted towards fully automated assays from vendors, e.g., Roche and Siemens Healthineers [14,33]. Another explanation for the underrepresentation of POC tests might be that the exception for these assays according to DIN EN ISO 15189 and therefore the rather low compliance of non-laboratory physicians regarding the proficiency testing regulations.

In this EQA, the majority of participants submitted results for determination of total SARS-CoV-2 IgG antibodies. The main clinical indication for total anti-SARS-CoV-2 Ig detection is the early detection phase. The ability of these assays to detect multiple antibody classes results in higher diagnostic sensitivity, in particular in the early phase of the immune response [34,35]. However, comparative investigations have proven that this is associated with a loss of diagnostic specificity [36].

For detection of total anti-SARS-CoV-2 IgG, the highest error rate was revealed, most of them attributed to the use of Roche. Overall, 234 results were submitted with Roche instruments for total anti-SARS-CoV-2 IgG. However, Roche does not offer any test for total antibody detection. Roche’s Elecsys^®^ SARS-CoV-2 antibody test detects all immunoglobulin classes, but is specific for antibodies to the nucleocapsid protein [37]. This can easily explain the high number of false results. In detail, the first and the fourth sample have been incorrectly reported as negative, as both samples were positive for anti-S-SARS-CoV-2 but negative for anti-N-SARS-CoV-2 and thus antibodies could not be detected by this assay. Hence, this high error rate can simply be attributed to a false clinical use of the tests by clinical laboratories that were not aware which antibodies are detected by the tests they use for clinical care. This highlights a significant shortcoming in the correct application of the assays and their interpretation that is of particular importance to differentiate between vaccination response and viral infection.

Spike-protein-specific antibodies were also determined by many participants, significantly more than participants who simultaneously determined nucleocapsid-specific antibodies. This is perplexing for a number of reasons. Although it has now been shown that vaccinations, primarily mRNA vaccines, can detect these antibodies at high levels of titer, interpretations of these quantitative values are not practically possible [38,39]. This assay alone cannot distinguish between an infection and the vaccination. This is only achieved by detecting antibodies against viral components that are not part of the vaccine [40]. This explains the importance of nucleocapsid-specific antibody detection as a method for detecting infection or vaccine breakthrough.

As already reported in prior EQA schemes [14,33,41], a high diversity of results reported was noted for the cut-off samples. This is also reflected in the data presented here. E.g., an error rate of 31% was seen for sample 2 for the determination of anti-N-SARS-CoV-2 IgG. Thus, all reported results (negative, positive, borderline) were considered correct in this EQA scheme. This could be explained by various assumptions. On the one hand, it could be an indicator of a lack of diagnostic sensitivity of the used assay. On the other hand, it could be interpreted as a deficit in the harmonization of results. In addition, other studies have indicated that in some cases laboratories use different thresholds, which may influence the results [14,42]. In this context, the International Federation of Clinical Chemistry (IFCC) already published recommendations in October 2020, specifically stating that results of antibody analyses must always be interpreted in their clinical context [43].

This study has clearly demonstrated that the validity and reliability of SARS-CoV-2 antibody detection is currently limited and the correct use of test systems and their interpretation in a clinical context needs to be further optimized. To achieve this, broader awareness of these shortcomings among manufacturers and laboratory experts is critical. The use of serological testing continues to increase in the context of longitudinal immunosurveillance, estimation of the need for further booster vaccinations, and monitoring of serological immune responses. Thus, quality assurance, especially with regard to the distinction between natural immunity and vaccination response, is becoming more important in order to achieve harmonization of test results and their interpretation in the future.

## 5. Limitations

A major limitation of this proficiency testing program was the lack of standardized reference material on a biological basis and a consensus on the reference method for the determination of anti-SARS CoV-2 antibodies [9]. Despite a steadily increasing number of commercially available and DIN EN ISO-certified reference materials, this statement is still valid [44,45]. This explains why the determination of target values was made by a panel of experts. This panel was only able to base the professional assessments on known clinical information, the results of various well-established immunoassays, and micro-VNT results. Another limitation of this study was the number of samples provided in this program, which was limited. A prerequisite for the evaluation of proficiency test results is that negative, positive and borderline samples (to evaluate assay performance) are provided. This could be used as an inspiration to send more samples in a new round of the proficiency testing program, which will have several advantages. One of the most important publications concerning EQA schemes and their significance was written by Miller et al. The authors stated that EQA schemes allow for the evaluation of the performance of individual laboratories and, if interchangeable samples are used, the state of standardization and harmonization between different measurement methods [46]. The authors distinguished between six categories of EQA schemes in descending quality of informative value. The proficiency test offered here fulfills all requirements for a category IV. This means that statements about the individual performance of each participating laboratory in general and in comparison to a peer-laboratory can be made. Furthermore, the variability between the different laboratories can be evaluated. The standardization and harmonization of the results versus the results of the participants can be compared [46]. An increase in the number of samples as mentioned above would make it technically possible to send a sample twice and thus obtain a statement about the Individual laboratory intra-lab CV, which would correspond to an upgrading of the EQA scheme to category III.

The addition of a larger number of samples in an EQA cycle would allow for distribution close to cut-off samples for each epitope. This would allow for significantly better assessments and comparisons of the diagnostic sensitivity in the borderline region of the various assays. According to Miller et al., the only way to achieve an even higher quality grade would be to use target-specific biological reference material. That would qualify the EQA as class II. However, these materials are currently not available.

## 6. Conclusions

In summary, the evaluation of this EQA data has highlighted various aspects of the pandemic situation. First of all, it should be emphasized that the test landscape remains heterogeneous but is consolidating more and more towards major vendors. Nevertheless, assays with limited clinical usefulness but seemingly high demand are being offered. Unfortunately, not all users are aware of the intended use of their methods, which is an application error and may lead to incorrect medical assessments. Regrettably, there have been no noticeable improvements in the standardization of methods.

Future research should be urgently used to focus on and improve the quality of the diagnostics being offered, especially as experience shows that the number of SARS-CoV-2 infections is expected to increase even more during the winter months. Current deficits should be overcome, and laboratories should be aware of their responsibility with regard to the reported results.

## Figures and Tables

**Table 1:** EQA samples results and target values.

	EQA Sample 1	EQA Sample 2	EQA Sample 3	EQA Sample 4
Roche Elecsys anti-N (COI/result)	negative	>250.00/reactive	negative	negative
Roche Elecsys anti-S (U/mL/result)	30.136/reactive	147.04/reactive	0.400/negative	<250.00 reactive
Euroimmun anti-N IgG (ratio/result)	0.37/negative	0.60/negative	0.18/negative	0.35/negative
Euroimmun anti-N IgS (ratio/result)	1.48/reactive	1.01/borderline	0.35/negative	5.05/reactive
VNT titer (wildtype) (titer/result)	<5/negative	<5/negative	<5/negative	10/positive
VNT titer (delta) (titer/result)	<5/negative	<5/negative	<5/negative	<5/negative
target value—Anti-SARS-CoV-2 IgG total	positive [borderline] *	positive [borderline]	negative	positive [borderline]
target value—Anti-N-SARS-CoV-2 IgG	negative	positive [borderline, negative]	negative	negative
target value—Anti-S-SARS-CoV-2 IgG	positive [borderline]	positive [borderline]	negative	positive [borderline]

* Results in square-brackets were considered conditionally correct.

**Table 2:** (**a**) Result summary by partial task and manufacturer for Anti-SARS-CoV-2 IgG total. (**b**) Result summary by partial task and manufacturer for Anti-S-SARS-CoV-2 IgG. (**c**) Result summary by partial task and manufacturer for Anti-N-SARS-CoV-2 IgG.

(**a**)											
**Task**	***n***	**%**	**Manufacturer**	**[*n*]**	**[%]**	**Correct [*n*]**	**[%]**	**Borderline [*n*]**	**[%]**	**Incorrect [*n*]**	**[%]**
**Anti-SARS-CoV-2 IgG total**	**624**	**49**	Bio-Rad	4	1	4	100				
			DiaSys	8	1	7	88	1	12		
			Abbott	52	8	40	77	1	2	11	21
			Beckman Coulter	16	3	12	75			4	25
			Euroimmun	96	15	88	92			8	8
			bioMerieux	4	1	4	100				
			IDS S.A.	4	1	3	75			1	25
			Roche	212	34	137	65	1	0	74	35
			lab developed assay	4	1	2	50			2	50
			Siemens Healthineers	24	4	24	100				
			Siemens Healthineers-Atellica	16	3	16	100				
			SERAMUN Diagnostica	4	1	4	100				
			DiaSorin	104	17	98	94			6	6
			Virotech Diagnostics	4	1	2	50			2	50
			others	24	4	16	67			8	33
			MöLAB	4	1	2	50	1	25	1	25
			nal von Minden	16	3	9	56	2	12	5	31
			AESKI.Diagnostics	4	1	3	75	1	25		
			Mikrogen	16	3	12	75			4	25
			Viramed Biotech	8	1	8	100				
value of correctness [average %]						79.45					
(**b**)											
**Task**	***n***	**%**	**Manufacturer**	**[*n*]**	**[%]**	**Correct [*n*]**	**[%]**	**Borderline [*n*]**	**[%]**	**Incorrect [*n*]**	**[%]**
**Anti-S-SARS-CoV-2 IgG**	**447**	**35**	Bio-Rad	4	1	4	100				
			Abbott	56	13	53	95			3	5
			Euroimmun	76	17	72	95			4	5
			bioMerieux	4	1	4	100				
			Roche	175	39	169	97			6	3
			Siemens Healthineers	8	2	8	100				
			IBL	4	1	4	100				
			Siemens Healthineers-Atellica	20	4	20	100				
			SERAMUN Diagnostica	4	1	4	100				
			Mediagnost	8	2	6	75	2	25		
			DiaSorin	36	8	36	100				
			others	16	4	11	69			5	31
			AESKI.Diagnostics	4	1	3	75	1	25		
			Mikrogen	8	2	8	100				
			Novatec	4	1	4	100				
			Bühlmann	4	1	4	100				
			Viramed Biotech	4	1	4	100				
			Virion/Serion	4	1	4	100				
			Snibe	8	2	8	100				
value of correctness [average %]						95.02					
(**c**)											
**Task**	***n***	**%**	**Manufacturer**	**[*n*]**	**[%]**	**Correct [*n*]**	**[%]**	**Borderline [*n*]**	**[%]**	**Incorrect [*n*]**	**[%]**
**Anti-N-SARS-CoV-2 IgG**	**204**	**16**	Bio-Rad	4	2	4	100				
			Abbott	44	22	10	23	1	2	33	75
			Euroimmun	20	10	4	20	1	5	15	75
			Roche	104	51	100	96			4	4
			SERAMUN Diagnostica	4	2	4	100				
			others	8	4	7	88			1	12
			AESKI.Diagnostics	4	2	3	75			1	25
			Mikrogen	12	6	12	100				
			Viramed Biotech	4	2	4	100				
value of correctness [average %]						78					

**Table 3:** (**a**) Task and manufacturer specific diagnostic sensitivity and specificity for Anti-SARS-CoV-2 IgG total. (**b**) Task and manufacturer specific diagnostic sensitivity and specificity for Anti-S-SARS-CoV-2 IgG. (**c**) Task and manufacturer specific diagnostic sensitivity and specificity for Anti-N-SARS-CoV-2 IgG (a/b/c: 95% confidence intervals were not shown due to the clarity of presentation.

(**a**)				
**Task**	**Manufacturer**	**Reported Results [*n*]**	**Diagnostic Sensitivity [%]**	**Diagnostic Specificity [%]**
**Anti-SARS-CoV-2 IgG total**	Abbott	52	72	100
	AESKI.Diagnostics	4	100	100
	Beckmann Coulter	16	75	100
	Bio Rad	4	100	100
	bioMeriéux	4	100	100
	DiaSorin	104	96	88
	DiaSys	8	100	100
	lab developed assay	4	33	100
	Euroimmun	96	92	92
	IDS S.A.	4	33	100
	MöLAB	4	66	100
	nal von Minden	16	59	100
	Roche	212	54	98
	SERAMUN Diagnostica	4	100	100
	Siemens Healthineers	24	100	100
	Siemens Healthineers Atellica	16	100	100
	Viramed Biotech	8	100	100
	Virotech Diagnostics	4	33	100
	others	24	56	100
Results: average [median]			77.32 [92]	98.84 [100]
(**b**)				
**Task**	**Manufacturer**	**Reported Results [*n*]**	**Diagnostic Sensitivity [%]**	**Diagnostic Specificity [%]**
**Anti-S-SARS-CoV-2 IgG**	Abbott	56	93	100
	AESKI.Diagnostics	4	100	100
	Bio Rad	4	100	100
	bioMeriéux	4	100	100
	Bühlmann	4	100	100
	DiaSorin	36	100	100
	Euroimmun	76	95	95
	IBL	4	100	100
	Mediagnost	8	100	100
	Mikrogen	8	100	100
	Novatec	4	100	100
	Roche	175	95	100
	SERAMUN Diagnostica	4	100	100
	Siemens Healthineers	8	100	100
	Siemens Healthineers Atellica	20	100	100
	Snibe	8	100	100
	Viramed Biotech	4	100	100
	Virion/Serion	4	100	100
	others	16	67	75
Results: average [median]			97.37 [100]	98.42 [100]
(**c**)				
**Task**	**Manufacturer**	**Reported Results [*n*]**	**Diagnostic Sensitivity [%]**	**Diagnostic Specificity [%]**
Anti-N-SARS-CoV- IgG *	Abbott	44	n.a.	100
	AESKI.Diagnostics	4	n.a.	100
	Bio Rad	4	n.a.	100
	Euroimmun	20	n.a.	100
	Mikrogen	12	n.a.	100
	Roche	104	n.a.	95
	SERAMUN Diagnostica	4	n.a.	100
	Viramed Biotech	4	n.a.	100
	others	8	n.a.	100
Results: average [median]			n.a.	99.44 [100]

* Sample 2 has been excluded.

**Table 4:** Passing rates.

Task	Participants [*n*]	Success/Passing Rate [%]	False Positive [%]	False Negative [%]
**Anti-SARS-CoV-2 IgG total**	**129**	64	3.7	20.2
**Anti-S-SARS-CoV-2 IgG**	**100**	96	1.8	0.9
**Anti-N-SARS-CoV-2 IgG**	**47**	90	n.a.	3.9

## Data Availability

Not applicable.
